# The Use of Buccal Fat Pad in the Treatment of Oral Submucous Fibrosis: A Newer Method

**DOI:** 10.1155/2012/935135

**Published:** 2012-05-15

**Authors:** K. Saravanan, Vinod Narayanan

**Affiliations:** ^1^Department of Oral and Maxillofacial Surgery, Vinayaka Missions Sankaracharya Dental College and Hospital, Salem 636308, India; ^2^Saveetha Dental College and Hospital, Saveetha University, Poonamalle High Road, Chennai 600077, India

## Abstract

*Purpose of the study*. This study was to evaluate the use of buccal fat pad as an interpositioning material in surgical management of oral sub mucous fibrosis. *Materials and methods*. A series of 8 cases with proven oral sub mucous fibrosis, with mouth opening less than 20 mm, involving the buccal mucosa were treated surgically in the Department of Oral and Maxillofacial Surgery, College of Dental Surgery, Saveetha University, Chennai. Pedicled buccal fat pad was used as an interpositioning material to cover the raw areas in the oral cavity after incision and release of fibrous bands. *Results*. In 8 patients, the range of pre operative mouth opening was 3–18 mm (mean 14 mm). As the result of the successful surgical procedure, the size of the intra operative mouth opening was ranged from 25–38 mm (mean 33.25 mm). The patients were discharged 5–7 days after the operation. The range of the mouth opening at this time was 25–36 mm (mean 30.63 mm). The results were evaluated using student's t test and found to be statistically significant. The pedicled grafts took up uneventfull.

## 1. Introduction

In 1952, Schwartz coined the term “atrophica idiopathica mucosa oris” to describe an oral fibrosing disease he discovered in 5 Indian women from Kenya [1]. Joshi [2] subsequently termed the condition oral submucous fibrosis (OSF) in 1953. Worldwide, estimates of oral submucous fibrosis indicate that 2.5 million people are affected, with most cases being concentrated on the Indian subcontinent, especially eastern and southern India [2]. Oral submucous fibrosis is widely prevalent in all age groups and across all socioeconomic strata in India. A sharp increase in the incidence of oral submucous fibrosis is noted after Pan Parag came into the market, and the incidence continues to increase. The majority of patients present with an intolerance to spicy food, rigidity of lip, tongue, and palate leading to varying degrees of limitation of opening of the mouth and tongue movements [3, 4]. 

The management of oral submucous fibrosis can be dealt with two broad categories: medical and surgical [5]. The medical management includes multiphase injections of hyaluronidase, hydrocortisone, placental extract, triamcinolone plus vitamin, and iron supplements. Intralesional steroids are probably satisfactory only in cases with minimal impairment of mouth opening. The medical management has been extensively reported in the review literature by Kerr et al. [6]. The surgical treatment is the method of choice in patients with marked limitation of mouth opening [7]. The following surgical modalities have been used: release of fibrous bands and covering of the raw areas with split thickness skin grafting, bilateral nasolabial flaps, palatal island flaps, tongue flaps, temporalis myotomy, and coronoidectomy [7]. 

Different surgical procedures described in the literature claim different success rates. The use of buccal fat pad as a grafting source is relatively recent. The buccal fat pad is a supple and lobulated mass, easily accessible, and mobilized. The buccal fat pad is mainly used to cover defects in the posterior maxilla, the buccal region, the hard palate, the soft palate, and the retromolar and pterygomandibular regions after tumor resections and oroantral communications after tooth extractions [8, 9].

So, the aim of this study is to evaluate the use of  buccal fat pad as an interpositioning material in surgical management of oral submucous fibrosis.

## 2. Materials and Methods

### 2.1. Materials

**Figure 1:**
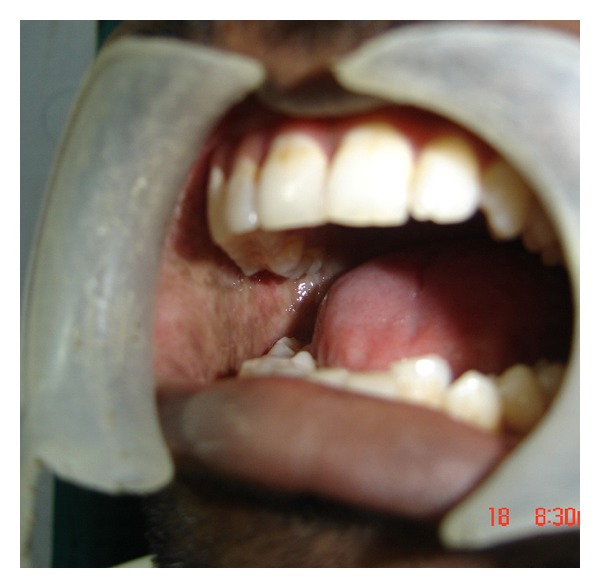
Bilateral oral submucous fibrosis (preop).

**Figure 2:**
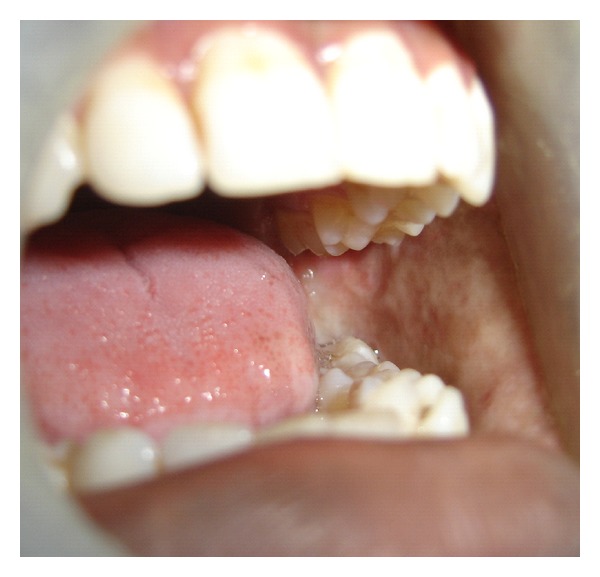
Bilateral oral submucous fibrosis (preop).

A series of 8 cases with proven oral submucous fibrosis, with mouth opening less than 20 mm, from 2007 to 2009 involving the buccal mucosa were treated surgically in the Department of Oral and Maxillofacial Surgery, College of Dental Surgery, Saveetha University, Chennai. Pedicled buccal fat pad was used as an interpositioning material to cover the raw areas in the oral cavity after incision and release of fibrous bands. All the patients had marked trismus. A detailed personal history with special reference to the tobacco habits was obtained from each patient. The preoperative interincisal distance was measured in every case with maximal mouth opening (Figures 1 and 2). Oral prophylaxis was done in all the cases preoperatively.

### 2.2. Methods

The surgeries were performed under general anesthesia with fibro-optic nasal intubation. The horizontal incision was placed along the occlusal plane with the surgical knife on the buccal mucosa away from Stenson's duct orifice. The incision was placed posteriorly to the pterygomandibular raphe or anterior pillar of fauces and at the anterior most point to the corner of the mouth. The fibrous bands were detected by digital palpation. Using fingers dissection made and fibrous bands were released. The wounds created were further freed by manipulation till no restrictions were felt. The mouth was then forced to open with the mouth gag to an acceptable range of approximately 35 mm. The coronoid process was approached from the wounds created or another incision placed along the external oblique ridge of the mandible. The coronoid process was exposed by subperiosteal dissection. Using bur the coronoid process was osteotomised and coronoidectomy was performed. The mouth was opened and the intraoperative opening was measured between upper and lower incisors. A mouth opening of 30–35 mm was considered to be the minimal acceptable opening in an adult. A small amount of the lesions were excised and sent for biopsy.

**Figure 3:**
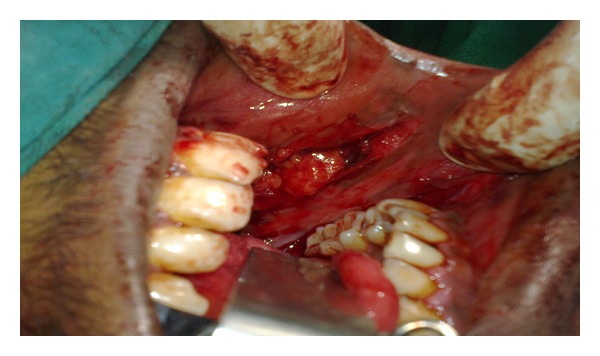
Incision and buccal fat pad exposed and harvested.

**Figure 4:**
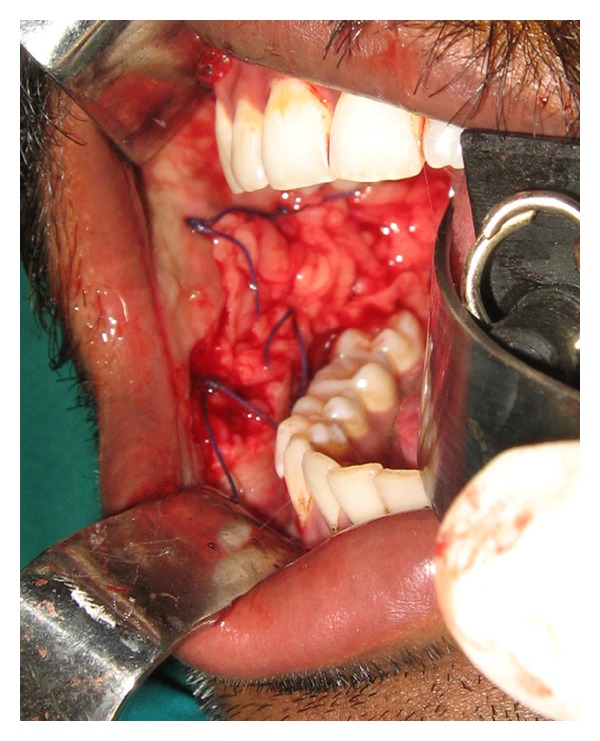
Buccal fat pad sutured (Intraop).

**Figure 5:**
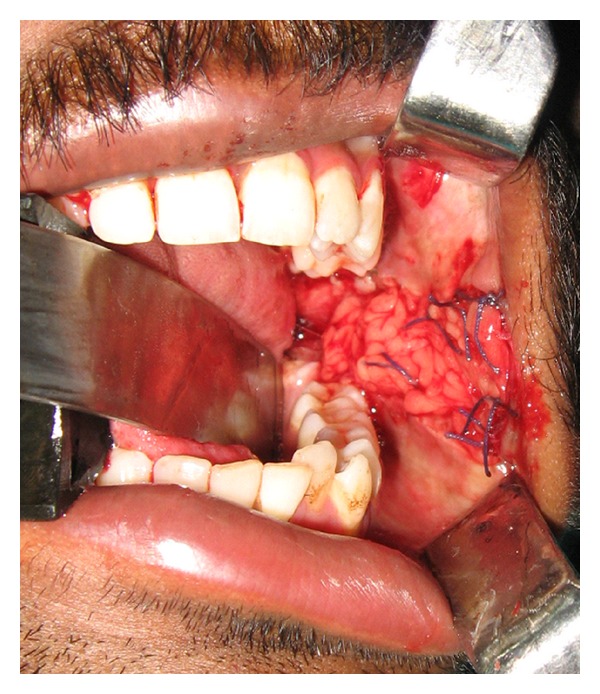
Buccal fat pad sutured (Intraop).

The buccal fat pad was approached on the posterior superior margin of the created buccal defect (Figure 3), that is, posterior to the zygomatic buttress. After blunt dissection, through the sub mucosa the buccal fat pad was mobilised gently until a significant amount was obtained to cover the defect without tension. This was done by using small artery forceps and gently letting out the buccal fat pad to the raw area. Pedicled buccal fat pad was used as a graft material to cover the areas. The buccal fat pad was secured in place by horizontal mattress sutures or simple interrupted sutures with 3/0 Vicryl. The same procedure was performed on the other side. The buccal fat pad covered the buccal defects posteriorly to the soft palate and anteriorly to the corners of the mouth (Figures 4 and 5).

All patients received prophylactic antibiotics. The regime was Ampicillin 1 gm IV before intubation. In selected cases where the mouth opening was severely restricted operatively, Ryle's tube was kept intraoperatively for feeding. All the patients had liquid diet postoperatively for at least 1 week. The patients were instructed to use 10 mL of Hexidine mouth rinse at 8 hourly. Mouth opening exercises were started postoperatively after 36 hours using Fergusson's mouth gag or wooden spatulas. The frequency of the exercises was carried out at 15–20 times thrice daily for at least 1 month for all patients. The patients were instructed to stop the tobacco habits strictly. All the patients were called for follow-up every month. Patients were asked for the status of signs and symptoms and a thorough clinical examination was done. The status of mouth opening was noted.

## 3. Results

**Figure 6:**
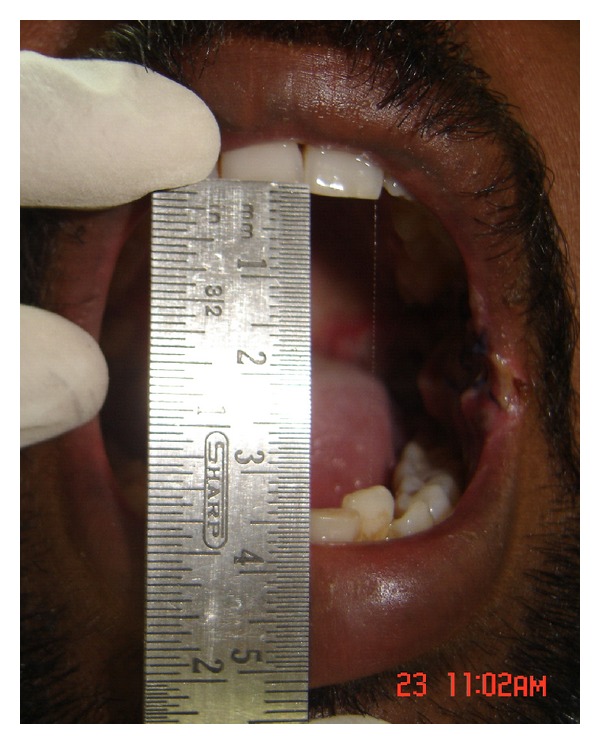
After 1 week.

**Figure 7:**
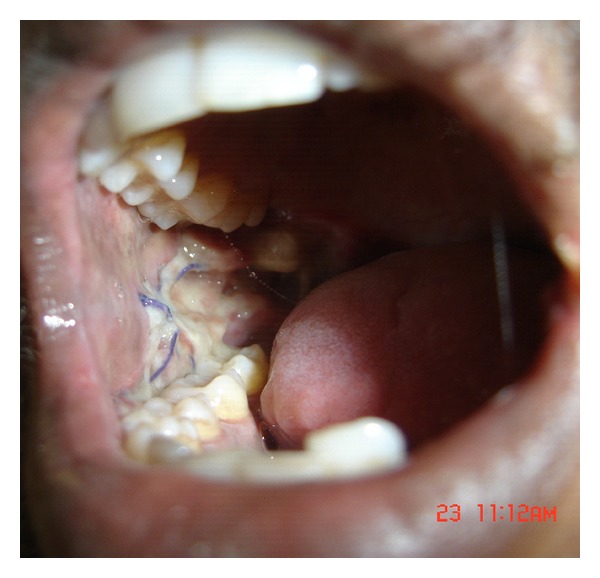
After 1 week.

**Figure 8:**
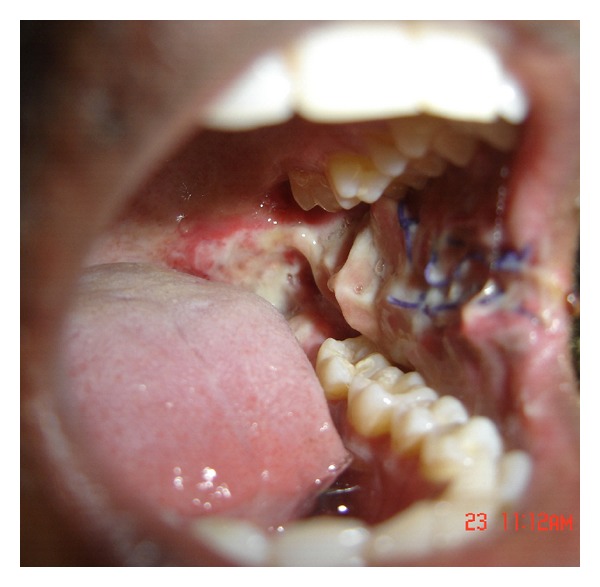
After 1 week.

**Figure 9:**
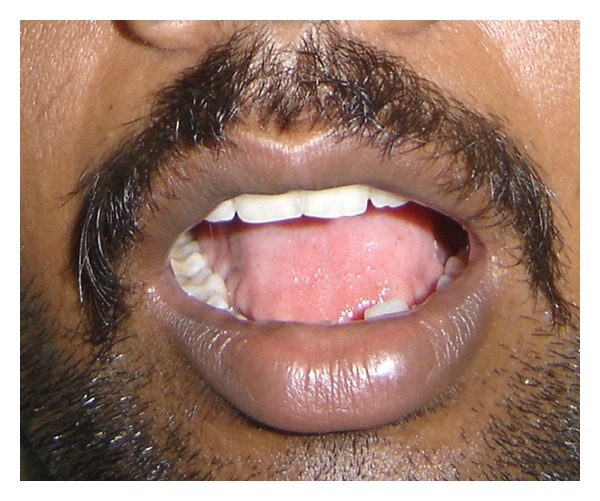
Preoperative picture.

**Figure 10:**
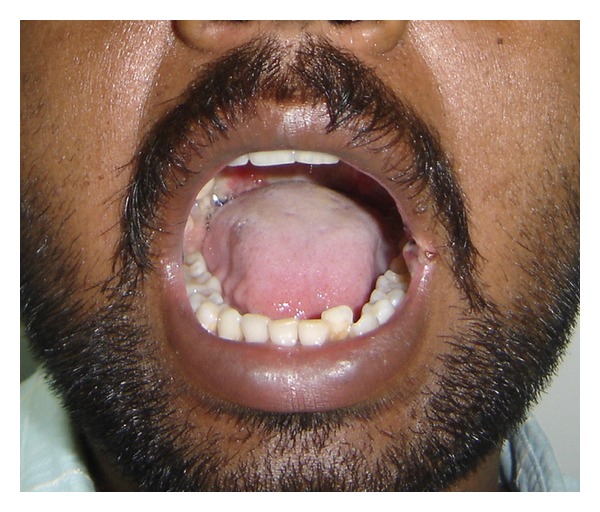
Postoperative picture.

The results were found to be satisfactory in all patients as shown in Tables 1 and 2. In 8 patients, the range of preoperative mouth opening was 3–18 mm (mean 14 mm) (Tables 1 and 2). As the result of the successful surgical procedure, the size of the intraoperative mouth opening was ranged from 25–38 mm (mean 33.25 mm). The patients were discharged 5–7 days after the operation (Figures 6, 7, and 8). The range of the mouth opening at this time was 25–36 mm (mean 30.63 mm). The pedicled grafts took up uneventfully and epithelialized in 3-4 weeks. One patient (case 5) failed to exercise several times daily and finally experienced a significant relapse. The other patients did cooperate and exercised daily and the results were satisfactory. The postoperative mouth opening after a follow-up period of 6 months was 18–35 mm (mean 30 mm) (Figures 9 and 10). The preoperative mouth opening was compared with intra- and each postoperative mouth opening using Students *t*-test and found to be statistically significant (*P* < 0.001 (99.9% sig)).

## 4. Discussion

Oral submucous fibrosis as an insidious chronic disease affecting any part of the oral cavity and sometimes pharynx, although occasionally preceded by and*∖*or associated with vesicle formation, is always associated with juxtaepithelial inflammatory reaction followed by fibroelastic changes in the lamina propria, with epithelial atrophy leading to stiffness of the oral mucosa causing trismus and difficulty in eating [10]. 

Most convincing evidence is derived from case-control studies [11] that estimated the odds ratios for areca nut use among oral submucous cases and a defined dose-dependant relationship between areca nut and the causation of the disease. Daily use appears to be more important than the duration of the habit. Both frequency and duration of chewing were important for the development of oral submucous fibrosis. The commercially freeze-dried products such as pan masala, Guthka, and mawa (areca and lime) have high concentrations of areca nut per chew and cause oral submucous fibrosis more rapidly than self-prepared conventional betel quid which contains smaller amounts of areca nut [11].

Early on, the histopathology [12] consists mostly of chronic inflammatory cells with an eosinophilic component infiltrating the subepithelial connective tissues. Older lesions demonstrate a reduced vascularity, reduced numbers of inflammatory cells, and dense bundles and sheets of collagen deposited immediately beneath the epithelium. The diffuse hyalinization of subepithelial stroma usually extends into the submucosal tissues, typically replacing the fatty and fibro vascular tissues. So the basic aim of the treatment modality has been relieving the symptoms which hamper function in the form of trismus, difficulty in mastication, deglutition, and speech.

The medical management has been extensively reported in the review literature by Kerr et al. [6]. They concluded with low-grade evidence to support recommendations for the nonsurgical management of oral submucous fibrosis.

The surgical procedures primarily aimed at the surgical elimination of fibrotic bands. The buccal fat pad is a supple and lobulated mass, easily accessible, and mobilized. Anatomically, the buccal fat pad is described as consisting of a central body and 4 extensions [8, 9]: buccal, pterygoid, pterygopalatine, superficial, and deep temporal. The main body is situated deeply along the posterior maxilla and upper fibers of the buccinator. The buccal extension lies superficially within the cheek and is mainly responsible for cheek fullness. The buccal extension and main body together constitute 55% to 70% of total weight. The blood supply of the buccal fat pad comes from 3 sources: maxillary artery (buccal and temporal branches), superficial temporal artery, and transverse facial artery. It is often encountered accidentally during maxillary orthognathic operations and there have been reports in children of spontaneous or traumatic herniation of buccal fat pad [9].

The easy mobilisation of the buccal fat pad and its excellent blood supply and minimal donor site morbidity makes it an ideal flap. The main advantages of buccal fat pad are ease of harvesting, simplicity, versatility, low rate of complications, as well as quick surgical technique. The operation can be performed in one incision, affecting neither appearance nor function of the area [12]. It has been used as pedicled graft in facial augmentation procedures, for the repair of persistent oroantral fistulas after dental extractions and in the reconstruction of small and medium size maxillary defects after resection of a tumor.

Canniff et al. [13] succeeded with split thickness skin graft following temporalis myotomy and coronoidectomy [14]. R. M. Borle and S. R. Borle [5] reported that skin graft was used to cover the defect after excision of fibrotic bands caused contracture during healing. The incidence of shrinkage, contracture, and rejection of the graft was found to be very high. Recurrences were common in the studies conducted by Khanna and Andrade [15]. The other limitation of split thickness skin graft is the morbidity associated with the donor site. Tongue flaps have been used to cover the buccal defects but were found to be bulky. Bilateral tongue flaps can cause severe dysphagia and carry the risk of postoperative aspiration. Bilateral full thickness nasolabial flap technique is the possible extraoral approach. Bilateral radial forearm flap has been used for the resection of buccal defects in oral submucous fibrosis [16].

The buccal fat pad by virtue of its anatomic position and excellent blood supply, the ease with which it can be accessed and mobilized, without any donor site morbidity, proved to be a logical, convenient, and reliable interpositioning material. The procedure considering the anatomic proximity of the donor and recipient site is not a lengthy one. The graft can be approached through the same buccal incision which was used to release the fibrotic bands. Should it fail, the consequences are not serious, as other options are open. The volume of buccal fat pad was found to be adequate in all cases, and it maintained its position as an interpositioning material postoperatively, similar to the findings of Yeh [17] and Sharma et al. [18]. Mehrotra et al. [19] compared the surgical treatment modalities in 100 patients using buccal fat pad, tongue flap, nasolabial flap, and split thickness graft and concluded that buccal fat pad graft was superior to all the other surgical procedures and that can be done even under local anaesthesia as a day care procedure. Postoperative healing was uneventful with no evidence of infection in all cases. In our study, the postoperative mouth opening range was 25–36 mm (mean 30.63 mm). Involvement in the physiologic functions like suppleness and elasticity of the buccal mucosa was seen on clinical examination. Symptoms such as burning sensation and intolerance to spices were eliminated in all the patients. This study showed the transferred buccal fat pad tissue was replaced by connective tissue postoperatively. All cases with more than 6 months follow-up were studied to correlate degree of mouth opening. It was observed that all cases show good postoperative mouth opening.

## 5. Conclusion

Considering the favorable results of this study with the advantages offered, buccal fat pad seems to be an appropriate interpositional graft in the surgical management of oral submucous fibrosis.

## Figures and Tables

**Table 1:** Results of treatment: mouth opening.

Case no.	Age/sex	Preop (mm)	Intraop (mm)	At discharge (mm)	After 1 month (mm)	After 6 months (mm)
1	27/M	15	35	30	33	33
2	30/M	14	33	30	32	30
3	23/M	12	30	27	29	28
4	37/M	18	33	29	31	30
5	45/F	3	25	25	20	18
6	32/M	18	37	35	35	34
7	27/M	17	38	36	35	35
8	32/M	15	35	33	33	32

**Table 2:** Student's *t*-test application in each variable.

	Mean	SD	SE	*t*	*P*
Preop	14.00	4.90	1.73	25.67	<0.001**
Intraop	33.25	4.17	1.47		
Preop	14.00	4.90	1.73	14.48	<0.001**
At discharge	30.63	3.82	1.35		
Preop	14.00	4.90	1.73	28.45	<0.001**
After one month	31.00	4.87	1.72		
Preop	14.00	4.90	1.73	23.48	<0.001**
After 6 months	30.00	5.37	1.90		

**Highly significant. Test *P* < 0.001 (99.9% sig).
